# Multisystem inflammatory syndrome in a fully vaccinated 18-year-old without known SARS-CoV-2 infection

**DOI:** 10.1186/s12969-022-00730-6

**Published:** 2022-09-01

**Authors:** Andy Liu, Alexa Love, Sophie Katz, Anna Patrick, David Parra, Natasha Halasa, Michael R. Miller

**Affiliations:** 1 Department of Pediatrics, Vanderbilt University Medical Center, Nashville, TN USA; 2 Division of Critical Care, Vanderbilt University Medical Center, Nashville, TN USA; 3 Monroe Carell Jr Children’s Hospital at Vanderbilt, 5121 DOT, 2200 Children’s Way, Nashville, TN 37232 USA; 4 Division of Infectious Diseases, Vanderbilt University Medical Center, Nashville, TN USA; 5 Division of Rheumatology, Vanderbilt University Medical Center, Nashville, TN USA; 6 Division of Cardiology, Vanderbilt University Medical Center, Nashville, TN USA

**Keywords:** MIS-C, COVID-19, SARS-CoV-2, Vaccination

## Abstract

**Background:**

Multisystem inflammatory syndrome in children (MIS-C) is a febrile syndrome that is observed in the pediatric population following severe acute respiratory syndrome 2 (SARS-CoV-2) infection. Vaccines have prevented or lessened the severity of the initial acute respiratory infection, while their effectiveness against severe MIS-C is just beginning to be reported.

**Case presentation:**

Here we report a fully vaccinated teenage female with no known history of SARS-CoV-2 infection who presented with shock and heart failure. Her presentation was initially thought secondary to a retropharyngeal abscess but was later identified as MIS-C after confirmed nucleocapsid antibody.

**Conclusions:**

Given the recent Omicron waves, the ongoing international outbreaks with evolving variants and the continued evolution of the COVID-19 pandemic, this case emphasizes the need to include MIS-C in the differential diagnosis, even in a fully vaccinated, previously healthy child.

## Background

A febrile syndrome that occurs in children weeks after the severe acute respiratory syndrome 2 (SARS-CoV-2) infection has been established as multisystem inflammatory syndrome in children (MIS-C) under 21 years of age [1, 2]. It is a systemic hyperinflammatory condition that presents with persistent fever, cardiac dysfunction, rash, generalized malaise, gastrointestinal symptoms, and not infrequently, hemodynamic instability and shock [2]. Neck pain, trismus, drooling, dysphagia, and retropharyngeal edema or inflammation found radiographically have been reported [3, 4]. As of August 1, 2022, there have been 8,798 cases of MIS-C, including 71 deaths, reported in the United States [5]. The incidence of MIS-C is approximately 300 children per 1 million SARS-CoV-2 infections, and it affects Black, Hispanic or Latino, and Asian or Pacific Islander children at disproportionally higher rates [5, 6]. The majority of MIS-C cases had mild or asymptomatic SARS-CoV-2 infections, and only about a quarter of children with MIS-C report preceding COVID-19 illnesses [7].

Vaccines have been developed to prevent or mitigate SARS-CoV-2 infection, and their effectiveness in reducing MIS-C is just now becoming apparent [8, 9], as few MIS-C cases were reported in fully vaccinated children only recently [10, 11]. A Centers for Disease Control and Prevention (CDC) report in January 2022 identified five fully vaccinated children who developed MIS-C between July and December 2021 [8]. Here we report a case of MIS-C in a fully vaccinated teenager who completed the Pfizer-BioNTech mRNA COVID-19 two-dose series six months prior to presentation. This case highlights the importance of considering MIS-C as part of the differential diagnosis early in the patient’s clinical presentation, even in a previously vaccinated patient may present with severe MIS-C.

## Case presentation

A previously healthy 18-year-old African American female presented to the emergency department (ED) with four days of fever, fatigue, headache, sore throat, odynophagia, abdominal pain, vomiting, and diarrhea. Prior to presentation to the ED, she had tested negative for *Group A Streptococcus*, influenza, mononucleosis, and SARS-CoV-2. She completed her Pfizer-BioNTech mRNA COVID-19 two-dose vaccine series six months ago, and had tested negative (rapid antigen) weekly for 2 months prior to presentation as part of routine screening at her college. She had no known symptomatic SARS-CoV-2 infection.

**Fig. 1:**
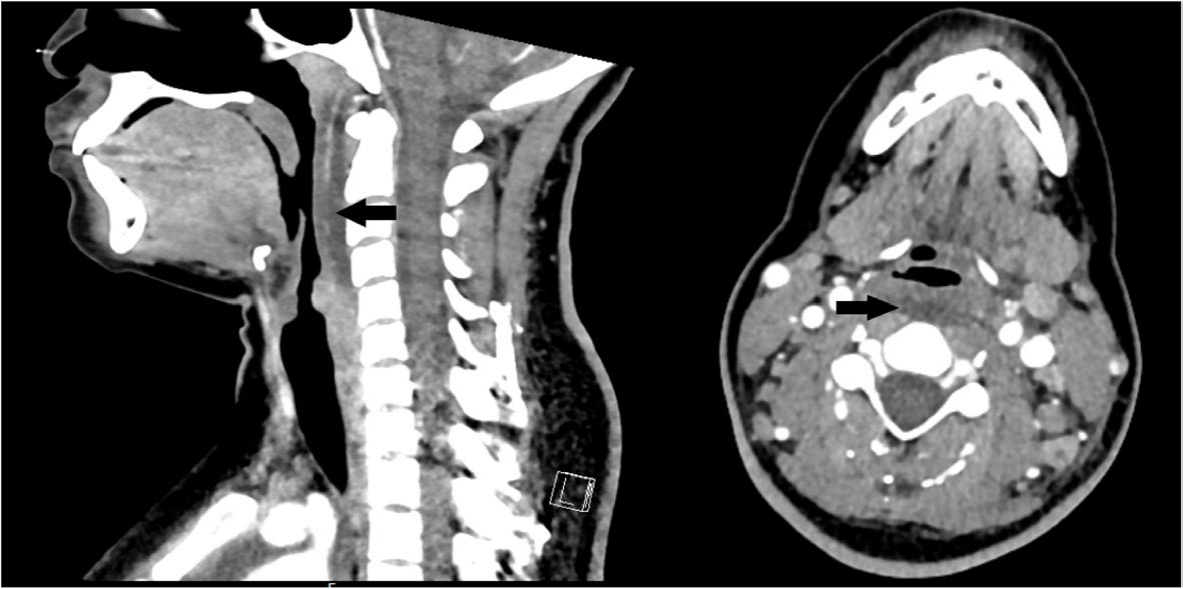
CT of the neck

In the ED, she was afebrile, tachycardic at 127 beats per minute and hypotensive with a blood pressure of 86/53 mmHg. On exam, she had tender bilateral cervical lymphadenopathy, trismus, fullness on the left side of her neck, and decreased range of neck motion. Pertinent negatives included no conjunctivitis or rash, and normal lung exam. Her initial workup was significant for leukocytosis, elevated CRP (Table 1), and negative SARS-CoV-2 PCR. Neck commuted tomography (CT) scan demonstrated a retropharyngeal fluid collection (6.1 × 2.7 × 0.5 cm) extending from C1 to C4 (Fig. 1). A diagnosis of retropharyngeal phlegmon was made and IV ampicillin-sulbactam was started in addition to a single dose of dexamethasone for airway edema.

**Table 1:** Lab results on admission, Hospital Day (HD) 1 (Transfer to PICU), HD 3 (first dose of IVIG), HD 5, and HD 7 (after IVIG)

Lab (Reference Range)	Admission	HD 1	HD 3	HD 5	HD 7
White Blood Cells (3.9–10.7 × 10^3^/mcL)	13.8	19.0	22.8	21.1	29.3
Hemoglobin (11.8–16.0 gm/dL)	12.0	9.4	9.5	8.6	9.0
Platelets (135–371 × 10^3^/mcL)	200	224	234	223	271
Neutrophils (absolute) (1.60–8.10 × 10^3^/mcL)	12.22	17.20	20.52	17.47	22.80
Lymphocytes (absolute) (1.10–3.50 × 10^3^/mcL)	0.48	1.50	0.57	2.58	1.03
C-Reactive Protein (0.1–1.7 mg/L)	400.7	352.4	415.0	327.5	118.6
Procalcitonin (≤ 0.24 ng/mL)	-	> 100.00	> 100.00	-	-
Sodium (136–145 mmol/L)	140	134	137	134	134
Creatinine (0.60–0.88 mg/dL)	1.04	0.94	0.66	0.8	0.56
Albumin (4.0–5.1 gm/dL)	4.1	3.2	2.7	2.8	2.5
Aspartate Aminotransferase (13–35 unit/L)	66	44	96	52	57
Alanine Aminotransferase (8–24 unit/L)	44	36	53	27	24
Bilirubin Total (0.1–0.8 mg/dL)	1.3	0.9	2.1	2.2	0.6
Lactate Whole Blood (0.5–2.2 mmol/L)	-	5.7	5.5	3.4	2.1
Troponin-I (≤ 0.03 ng/mL)	-	-	0.22	0.18	-
Blood Culture	-	No Growth	-	-	-
Urine Culture	-	No Growth	-	-	-
COVID-19 IgG (Negative)	-	-	Positive	-	-

Table 1. Laboratory findings relative to MIS-C targeted interventions

CT of the neck demonstrating a retropharyngeal fluid collection presumed to be the source for the patient’s original presentation. Arrows indicate sagittal view of retropharyngeal fluid collection measuring 6.1 cm, from C1 to C4 (left) and a coronal view of retropharyngeal fluid collection measuring 2.7 × 0.5 cm (right).

On the day after admission [Hospital Day (HD) 1], she was tachycardic to the 150s, hypoxemic requiring supplemental oxygen by nasal cannula, and developed fluid refractory hypotension with blood pressures of 70s/50s mmHg. She was transferred to the pediatric intensive care unit (PICU) and started on norepinephrine for presumed septic shock. Labs were notable for elevated procalcitonin, lactate and troponin, in addition to persistent leukocytosis (Table 1). Antibiotics were broadened from ampicillin-sulbactam to vancomycin, clindamycin, and levofloxacin. Echocardiogram (ECHO) showed markedly depressed biventricular function with a left ventricular ejection fraction (LVEF) of 22%. Norepinephrine was transitioned to epinephrine for inotropic support and dobutamine was added. Respiratory support was escalated to high flow nasal cannula and briefly to bilevel positive airway pressure due to hypoxemia and increased work of breathing.

On HD 2, she remained febrile, tachycardic, and tachypneic despite a repeat neck CT that demonstrated moderate decrease in the retropharyngeal fluid collection. Repeat ECHO demonstrated improvement in cardiac function with LVEF of 42% while on vasopressors. On HD 3, her antibiotics were broadened to vancomycin, cefepime and metronidazole to cover for indolent infection given her lack of clinical improvement and persistently elevated CRP to 415 mg/L.

**Fig. 2:**
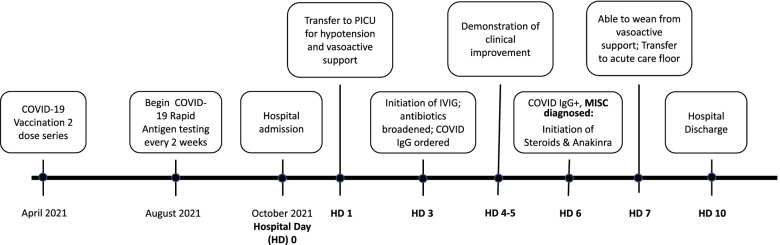
Key events timeline

Given her continued critical illness on multiple vasopressors and broad-spectrum antibiotics, MIS-C was considered in the differential diagnosis, and the decision was made to administer intravenous immune globulin (IVIG). She received IVIG on HD 3 and received a total of 2 g/kg of IVIG; given her heart failure and initial LVEF, it was administered over the course of four days (HD 3—HD 6). After initiation of IVIG, she began to defervesce (afebrile for 24 h by HD 5), and her CRP and lactate improved (Table 1). Her ventricular function improved on ECHO (LVEF 55% on HD 4 on vasopressors). On HD 6, her COVID-19 IgG (drawn prior to IVIG treatment) to nucleocapsid protein returned positive, and the diagnosis of MIS-C was made. Anti-inflammatory treatment for MIS-C was initiated with intravenous methylprednisolone and subcutaneous anakinra. On HD 7, she was weaned from vasopressor support and transferred back to the to the acute care floor where she continued to recover. ECHO on HD 9 demonstrated normal biventricular function. She was discharged home on HD 10 with subcutaneous anakinra and continued steroids (Fig. 2).

A timeline demonstrating key events in the chronology of the patient’s presentation and subsequent interventions. PICU—pediatric intensive care unit; IVIG, intravenous immunoglobulin; MIS-C, multisystemic inflammatory syndrome in children.

She had an ECHO at her follow up appointment with Cardiology 12 days after discharge which showed an LVEF of 59% with no valve or coronary abnormalities. She completed anakinra and prednisone tapers per instructions by Pediatric Rheumatology. Cardiac magnetic resonance imaging (MRI) was obtained three weeks after discharge with normal EF without evidence of myocardial inflammation by parametric mapping and there was no late gadolinium enhancement of the myocardium. Per patient report she has made full recovery with no sequelae.

## Discussion and conclusion

The CDC issued a Health Advisory on May 14, 2020, that defined a febrile syndrome following SARS-CoV-2 infection as MIS-C and provided case criteria [12]. It includes a positive test for current or recent SARS-CoV-2 infection or exposure to a suspected or confirmed COVID-19 case within the 4 weeks prior to the onset of symptoms, in addition to clinical syndrome and laboratory findings, and no alternative plausible diagnoses. Here we report a case of MIS-C in a teenage female who was fully immunized six months prior to presentation, had tested negative weekly at her college for the two months prior to presentation, and had no known COVID-19 exposure or symptomatic infection. There have been few reports of MIS-C in fully vaccinated children as of December 2021, one child with sickle cell disease and one with no significant medical history reported [10, 11]. An early release of a CDC report in January 2022 identified five fully vaccinated children who developed MIS-C between July and December 2021, estimating 91% vaccine effectiveness against MIS-C [8]. Only one of the aforementioned cases required vasoactives, and our patient was in both severe cardiogenic shock and hypoxemic respiratory failure. Our patient presented with signs and symptoms, as well as laboratory and radiographic findings of MIS-C; however, her vaccination status and negative surveillance tests reduced our pre-test probability. Her CT scan finding of retropharyngeal fluid collection led to septic shock secondary to a retropharyngeal abscess as the alternative diagnosis, and she was treated accordingly. Cases of MIS-C with retropharyngeal edema and/or phlegmon have been reported, though they are neither common nor defining features of MIS-C [3, 4]. We weighed the risks and benefits of MIS-C treatments, and decided to trial IVIG but hold methylprednisolone for concerns of serious bacterial infections until the confirmation of her past COVID-19 infection. Antibodies against SARS-CoV-2 viral spike proteins are elicited by vaccines and confer immunity, whereas COVID-19 antibody tests that detect nucleocapsid proteins of the SARS-CoV-2 virus indicate natural infection [13]. Her COVID-19 IgG to nucleocapsid protein later returned positive, meeting the missing criterium of positive serology and confirming the diagnosis of MIS-C. Once she was started on treatment for MIS-C, she progressed to full recovery.

MIS-C is well described and defined [1, 2], but the role for COVID-19 vaccines in preventing MIS-C is assumed but not clearly established [8–11]. Her vaccination did not cause her MIS-C [14] as she was far out from her vaccination, and she tested positive for COVID-19 IgG antibody to nucleocapsid protein. Our patient likely had a breakthrough infection that went undetected. Her case is unique in the degree of shock she presented with secondary to her MIS-C despite of vaccination. Her delay in diagnosis led to unnecessary antibiotic exposure, prolongation of her ICU length of stay, and put her at increased risk for ICU delirium. Given the recent Omicron waves and the ongoing evolution of a dynamic international pandemic with evolving variants, providers should have clinical suspicion for MIS-C when clinical signs and symptoms are consistent with the diagnosis, even in a previously healthy, fully vaccinated child. It is worth consideration that her cardiac MRI performed following her discharge showed near normal function and no residual inflammation. Further investigation into whether vaccination provides additional prevention against long-term sequelae from MIS-C is warranted. Given the broad range of providers (general practitioner, emergency medicine, hospitalist medicine, and critical care) that are involved in the care of these children, her presentation, and patients similar to her are critical for the field to be aware of.

## Data Availability

Data sharing is not applicable to this article as no datasets were generated or analyzed during the current study.

## Abbreviations

CDC: Centers for Disease Control and Prevention
CRP: C-reactive protein
ECHO: Echocardiogram
ED: Emergency department
EF: Ejection fraction
HD: Hospital day
IVIG: Intravenous immunoglobulin
MIS-C: Multisystem inflammatory syndrome in children
PICU: Pediatric intensive care unit
SARS-CoV-2: Severe acute respiratory syndrome 2
